# Erythropoiesis-stimulating agent resistance is associated with diabetic kidney disease but not with diabetes: a post hoc analysis of the BRIGHTEN study

**DOI:** 10.1007/s10157-026-02881-2

**Published:** 2026-05-18

**Authors:** Ryousuke Aoki, Keiichi Matsuzaki, Hitoshi Suzuki, Tatsuo Kagimura, Tadashi Sofue, Takashi Wada, Ichiei Narita, Yusuke Suzuki

**Affiliations:** 1 Department of Nephrology, Juntendo University Faculty of Medicine, 2-1-1 Hongo, Bunkyo-ku, Tokyo, 113-8421 Japan; 2 Department of Public Health, Kitasato University School of Medicine, Kanagawa, Japan; 3 Department of Nephrology, Juntendo University Urayasu Hospital, Chiba, Japan; 4 Translational Research Center for Medical Innovation , Foundation for Biomedical Research and Innovation at Kobe, Kobe, Japan; 5 Department of Cardiorenal and Cerebrovascular Medicine, Faculty of Medicine , Kagawa University, Takamatsu, Japan; 6 Department of Nephrology and Rheumatology, Kanazawa University, Kanazawa, Japan; 7 Division of Clinical Nephrology and Rheumatology, Kidney Research Center, Niigata University Graduate School of Medical and Dental Sciences, Niigata, Japan

**Keywords:** Diabetic kidney disease, Proteinuria, Renal anemia

## Abstract

**Background:**

Erythropoiesis-stimulating agent (ESA) resistance in chronic kidney disease (CKD) is influenced by various factors, including diabetes. Although diabetes has been linked to ESA resistance, the BRIGHTEN study in Japan found no direct correlation. This study aimed to identify factors contributing to ESA resistance in patients with diabetes by analyzing the diabetic kidney disease (DKD) status and CKD stage.

**Methods:**

A post hoc analysis was conducted using data from the BRIGHTEN study, a multicenter prospective observational trial investigating ESA resistance in patients with non-dialysis-dependent CKD. Patients were categorized based on diabetes status (DM vs. non-DM) and further stratified by CKD stage and presence of DKD. ESA resistance was assessed using the erythropoietin resistance index.

**Results:**

No significant differences in ESA resistance were observed between the DM and non-DM groups. However, among patients with diabetes, those with DKD exhibited higher ESA resistance and required higher darbepoetin alfa (DA) dosages than did those with non-DKD nephropathy (NDKD-DM). ESA resistance and DA dosage increased with CKD progression, with notable differences in CKD stage G4 between the DKD and NDKD-DM groups.

**Conclusion:**

Although diabetes alone was not associated with ESA resistance, patients with DKD exhibited higher resistance, suggesting that ESA resistance is more closely linked to DKD than to diabetes itself. These findings highlight the role of proteinuria and inflammation in the ESA response, emphasizing the need for individualized anemia management strategies based on nephropathy type.

**Supplementary Information:**

The online version contains supplementary material available at 10.1007/s10157-026-02881-2.

## Introduction

The mechanisms of anemia in chronic kidney disease (CKD) include a shortened red blood cell lifespan, reduced erythropoietin (EPO) production, increased erythropoiesis-stimulating agent (ESA) resistance, and impaired red blood cell production due to the accumulation of uremic metabolites [1].

Some studies suggest that a relative deficiency of EPO may develop in patients with diabetes without any evidence of renal damage and that synthetic EPO administration could help treat this form of anemia [2]. In addition, anemia in patients with diabetes is thought to result from decreased absolute reticulocyte ratio due to impaired red blood cell production caused by EPO deficiency, as well as increased ESA resistance due to abnormalities in the red blood cell membrane structure [2–4].

The “oBservational clinical Research In chronic kidney disease patients with renal anemia: renal proGnosis in patients with Hyporesponsive anemia To Erythropoiesis-stimulating agents, darbepoetiN alfa (BRIGHTEN)” study is a multicenter, prospective, observational study of patients with non-dialysis CKD with renal anemia [5]. The BRIGHTEN study aimed to clarify the relationship between markers and factors associated with decreased ESA responsiveness and the occurrence of renal and cardiovascular events [5, 6]. In a study investigating the initial responsiveness to darbepoetin alfa (DA) and its contributing factors, high hemoglobin (Hb), elevated C-reactive protein (CRP), increased N-terminal pro-brain natriuretic peptide, and a high urinary protein-creatinine ratio (UPCR) were significantly associated with a poor initial response to DA [7]. However, the initial study did not identify an association between diabetes and ESA resistance [7], suggesting that other factors contribute to ESA resistance in patients with diabetes. Because poor ESA reactivity is associated with increased mortality [8], it is crucial to identify the factors contributing to ESA resistance in advance and provide appropriate treatment.

Based on these findings, we conducted a post hoc analysis using data from the initial study. This study aimed to determine the factors related to ESA resistance by performing a detailed analysis of patients with diabetes according to the presence or absence of diabetic nephropathy and the CKD stage.

## Methods

### Study design

The BRIGHTEN study is a multicenter, prospective, observational study conducted to evaluate ESA resistance to DA for the treatment of anemia in non-dialysis-dependent CKD in a clinical setting [8]. Briefly, DA was administered at 30 µg within the first 8 weeks of enrollment. If hemoglobin levels showed improvement every 2 weeks, DA was subsequently administered once every 4 weeks. DA and iron supplementation were adjusted to maintain an Hb level > 11 g/dL according to the guidelines, and the patients were observed for at least 96 weeks from the start of DA treatment. The study protocol was approved by the Institutional Review Board of Niigata University (No. MH26-004) and the Institutional Review Board of Juntendo University Hospital (No. C23-0036). This research was conducted in accordance with the principles of the Declaration of Helsinki and the Ethical Guidelines on Clinical Studies of the Ministry of Health, Labour, and Welfare of Japan. Written informed consent was obtained from all the participants.

### Study population

Of The 1980 patients enrolled in 168 facilities, 285 were excluded mainly because of the lack of Hb values at 0 and/or 12 weeks (84 ± 14 days). Finally, 1405 patients were included in the present analysis. The patients’ medical histories were carefully documented through interviews and confirmed using clinical records, including prescribed medications, imaging diagnostics, and laboratory data. When available, pathological diagnosis was used to determine the cause of CKD. Otherwise, diabetic kidney disease (DKD), non-DKD, and diabetic nephropathy (DN) were classified based on clinical diagnoses (Supplementary Table 1) [9–12]. The patients were assigned to two groups according to whether or not they had diabetes: DM (*n* = 609) or non-DM (*n* = 796). Patients with DM were assigned to two groups according to the cause of CKD: DKD (*n* = 391) and non-DKD nephropathy (NDKD-DM) (*n* = 218). NDKD-DM refers to other groups of nephropathies besides DKD that are complicated by diabetes. Additionally, each group was categorized according to the estimated glomerular filtration rate (eGFR) value at enrollment as follows: CKD G3 (30 ≦ eGFR < 59), CKD G4 (15 ≦ eGFR < 29), and CKD G5 (eGFR < 15).

### Outcomes and response index to DA

For the evaluation of ESA reactivity, the following formula (erythropoietin resistance index; ERI [12]) was used initially, as in the primary study [5].

$$ERI - 1B = ~\frac{{Dosage~of~DA~at~12~weeks~\left( {\mu g} \right)}}{{Concentration~of~Hb~\left( {g/dL} \right)~at~12~weeks}}$$

This study analyzed temporal changes in ERI-1B and differences in DA dosage among groups.

### Statistical analysis

Baseline characteristics were reported as means ± standard deviation, medians (interquartile range), or numbers (percentages). Differences between the groups were analyzed using analysis of variance for continuous values, the Kruskal–Wallis test for ordinal variables, and the Fisher’s exact test or, where appropriate, the X^2^ test for frequency distributions. Regarding the predictors of the initial response to DA, the association between ERI-1B and DA dosage was first investigated. Longitudinal changes in ERI-1B and DA dosage after Week 12 were analyzed using a mixed-effects model for repeated measures (MMRM). Adjusted were made by adding Age, Sex, Body Weight, Prevalence of smoking and prevalence of malignancy as covariates to the model. Adjusted means were estimated as least-squares means with 95% confidence intervals. All analyses were performed using SAS version 9.4 (SAS Institute, Cary, NC, USA) and R 4.3.1. Statistical significance was set at *P* < 0.05.

## Results

### Baseline characteristics of the DM versus non-DM group

The clinical characteristics of the DM and non-DM groups are presented in Table 1. In the DM group, DN was the leading cause of CKD (64.2%), followed by nephrosclerosis (10.8%) and chronic glomerulonephritis (10.8%). In the non-DM group, DN was not included, and 35.6% of patients had nephrosclerosis, while 30.5% had chronic glomerulonephritis. Patients with DM were younger and had a higher male-to-female ratio, a greater proportion of current smokers, a higher UPCR, and an elevated glycated hemoglobin (HbA1c) level than those in the non-DM group. Furthermore, patients with DM had higher BMI and SBP than those without DM. Patients without DM were less likely to take a renin-angiotensin system (RAS) inhibitor than those with DKD. In contrast, the percentage of iron supplementation did not differ between the two groups. No significant differences were observed between the two groups in baseline serum creatinine (Cr), eGFR, or ferritin levels.

**Table 1:** Clinical characteristics and laboratory findings of the DM and non-DM groups

	DM (DKD + NDKD-DM) (*n* = 609)	non-DM (*n* = 796)	*P-value*
Age, year	69.1 ± 11.3	70.7 ± 12.3	0.011*
Male sex (%)	64.9	53.3	< 0.001*
BMI, kg/m^2^	24.5 ± 4.38	22.2 ± 3.63	< 0.001*
BW, kg	62.9 ± 13.3	55.7 ± 11.3	< 0.001*
Etiology of chronic kidney disease			< 0.001*
Diabetic nephropathy (%)	64.2	0	
Nephrosclerosis (%)	10.8	35.6	
Chronic glomerulonephritis (%)	10.8	30.5	
Others (%)	14.1	33.9	
Current smoker (%)	14.9	7.2	< 0.001*
Medication			
RAS inhibitors (%)	71.1	64.1	0.006*
Iron supplementation (%)	15.8	15.3	0.881
Past history			
HT (%)	98.0	94.3	0.001*
DL (%)	67.5	51.3	< 0.001*
Malignant tumor (%)	12.6	12.6	1
SBP, mmHg	135.9 ± 19.6	133.1 ± 18.4	0.006*
DBP, mmHg	69.7 ± 12.0	72.5 ± 12.6	< 0.001*
Hb, g/dL	9.7 ± 0.9	9.8 ± 0.9	0.218
Serum creatinine, mg/dL	2.89 ± 1.30	2.89 ± 1.41	0.951
eGFR, mL/min/1.73m^2^	20.2 ± 9.7	19.6 ± 9.5	0.223
Alb, g/dL	3.60 ± 0.55	3.81 ± 0.47	< 0.001*
HbA1c (NGSP), %	6.38 ± 0.98	5.58 ± 0.37	< 0.001*
Ferritin, ng/mL	145.0 ± 143.4	126.5 ± 121.4	0.010*
TSAT, %	25.8 ± 9.9	27.3 ± 9.7	0.003*
NT-proBNP (pg/mL)	617.00 (274.00–1367.50)	457.00 (231.00–1030.00)	< 0.001*
UPCR, g/gCr	3.24 ± 3.63	1.70 ± 2.48	< 0.001*
hsCRP, ng/mL	672.50 (261.00–1997.50)	537.00 (199.00–1510.00)	0.016*

Clinical characteristics and laboratory findings of the DM and non-DM groups. Categorical variables are presented as numbers (percentages). Data are presented as mean ± standard deviation, median (25th and 75th percentile), or percentage

*Alb* serum albumin, *DM* Diabetes mellitus, *DKD* diabetic kidney disease, *NDKD-DM* non-DKD nephropathies with Diabetes mellitus, *BMI* body mass index, *BW* body weight, *eGFR* estimated glomerular filtration rate, *Hb* hemoglobin, *HbA1c* glycated hemoglobin, *RAS* renin-angiotensin system, *HT* hyper tension, *DL* dyslipidemia, *SBP* systolic blood pressure, *DBP* diastolic blood pressure, *NT-proBNP* N-terminal pro-brain natriuretic peptide, *UPCR* urine total protein to creatinine ratio, *hsCRP* high-sensitivity CRP

* P < 0.05

**Fig. 1:**
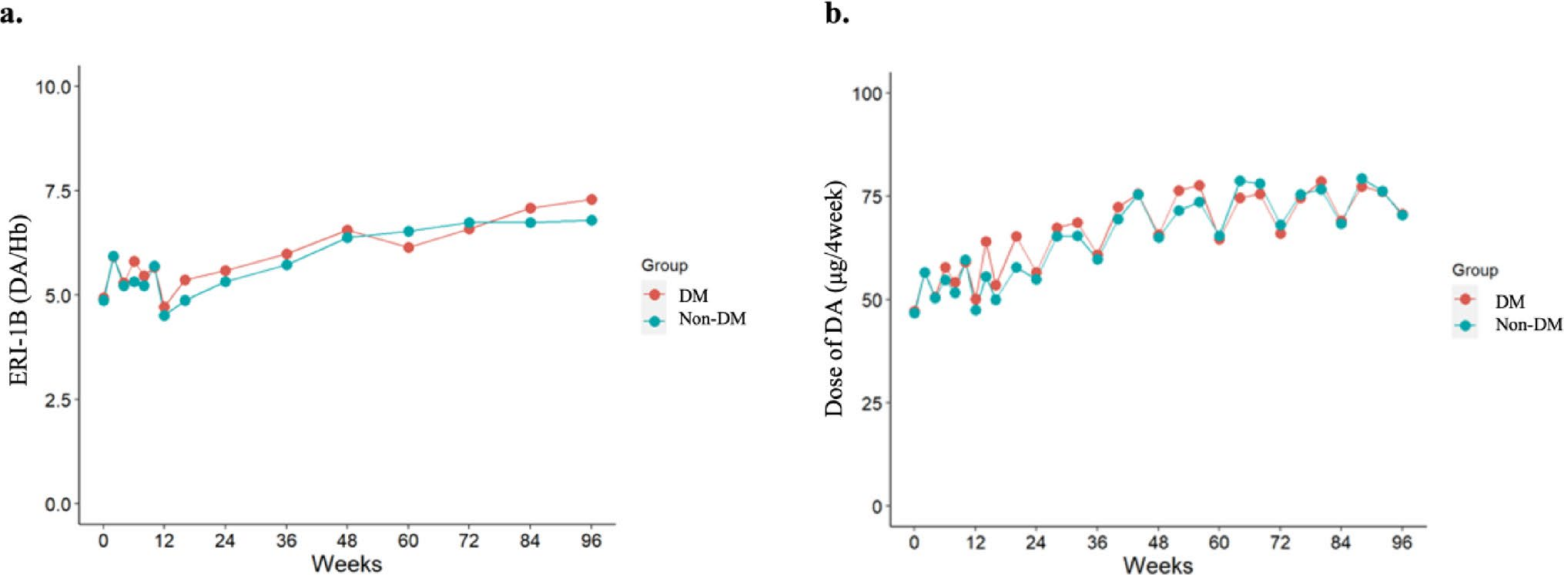
Changes in ERI-1B and DA dosage between the DM and non-DM groups Change in **a** ERI-1B levels and **b** dosage of DA administered in the DM and non-DM groups *DM* Diabetes mellitus, *DA* darbepoetin alfa, *ERI* erythropoietin resistance index, *ESA* erythropoiesis-stimulating agent

The changes in ERI-1B and DA dosages over time are shown in Fig. 1. No significant time-dependent differences were observed between the DM and non-DM groups in ERI-1B variation or DA dosage.

### The DKD group had higher ESA resistance than did the NDKD-DM group

The DKD group included only patients with DN, whereas the NDKD-DM group included 30.3% of patients with nephrosclerosis and 30.3% with chronic glomerulonephritis (Table 2). The DKD group was younger and had a higher proportion of men. However, no significant difference was observed in BMI or the proportion of patients taking RAS inhibitors between the two groups. The UPCR at baseline was 3.72 ± 3.64 g/gCr in the DKD group and 2.37 ± 3.46 g/gCr in the NDKD-DM group.

**Table 2:** Clinical characteristics and laboratory findings of the DKD and NDKD-DM groups

	DKD (*n* = 391)	NDKD-DM (*n* = 218)	*P-value*
Age, year	67.2 ± 11.7	72.5 ± 9.61	< 0.001*
Male sex (%)	68.3	58.7	0.022*
BMI, kg/m^2^	24.8 ± 4.5	24.1 ± 4.1	0.062
BW, kg	64.4 ± 14.0	60.3 ± 11.6	< 0.001*
Etiology of chronic kidney disease			< 0.001*
Diabetic nephropathy (%)	100.0	0.0	
Nephrosclerosis (%)	0.0	30.3	
Chronic glomerulonephritis (%)	0.0	30.3	
Others (%)	0.0	39.4	
Current smoker (%)	18.2	9.2	0.003*
Medication			
RAS inhibitors (%)	72.9	67.9	0.226
Iron supplementation (%)	16.4	14.7	0.665
Past history			
HT (%)	98.0	98.2	1
DL (%)	69.8	63.3	0.120
Malignant tumor (%)	9.0	19.3	< 0.001*
SBP, mmHg	137.4 ± 19.6	133.2 ± 19.3	0.013*
DBP, mmHg	70.3 ± 12.2	68.7 ± 11.5	0.100
Hb, g/dL	9.7 ± 0.9	9.8 ± 0.91	0.042*
Serum creatinine, mg/dL	2.99 ± 1.30	2.71 ± 1.27	0.010*
eGFR, mL/min/1.73m^2^	19.8 ± 9.7	21.0 ± 9.7	0.126
Alb, g/dL	3.56 ± 0.56	3.69 ± 0.53	0.004*
HbA1c (NGSP), %	6.46 ± 1.09	6.23 ± 0.71	0.011*
Ferritin, ng/mL	144.4 ± 140.1	146.2 ± 149.3	0.885
TSAT, %	25.7 ± 9.3	25.9 ± 10.7	0.752
NT-proBNP (pg/mL)	632.50 (279.25–1210.00)	573.00 (265.25–1330.00)	0.733
UPCR, g/gCr	3.72 ± 3.64	2.37 ± 3.46	< 0.001*
hsCRP, ng/mL	573.00 (229.00–1907.50)	917.00 (285.25–2120.00)	0.025*

Clinical characteristics and laboratory findings of the DKD and NDKD-DM groups. Categorical variables are presented as numbers (percentages). Data are presented as mean ± standard deviation, median (25th and 75th percentile), or percentage

*DKD* diabetic kidney disease, *NDKD-DM* non-DKD nephropathies with Diabetes mellitus, *BMI* body mass index, *BW* body weight, *eGFR* estimated glomerular filtration rate, *Hb* hemoglobin, *HbA1c* glycated hemoglobin, *RAS* renin-angiotensin system, *HT* hyper tension, *DL* dyslipidemia, *SBP* systolic blood pressure, *DBP* diastolic blood pressure, *NT-proBNP* N-terminal pro-brain natriuretic peptide, *UPCR* urine total protein to creatinine ratio, *hsCRP* high-sensitivity CRP

* P < 0.05

**Fig. 2:**
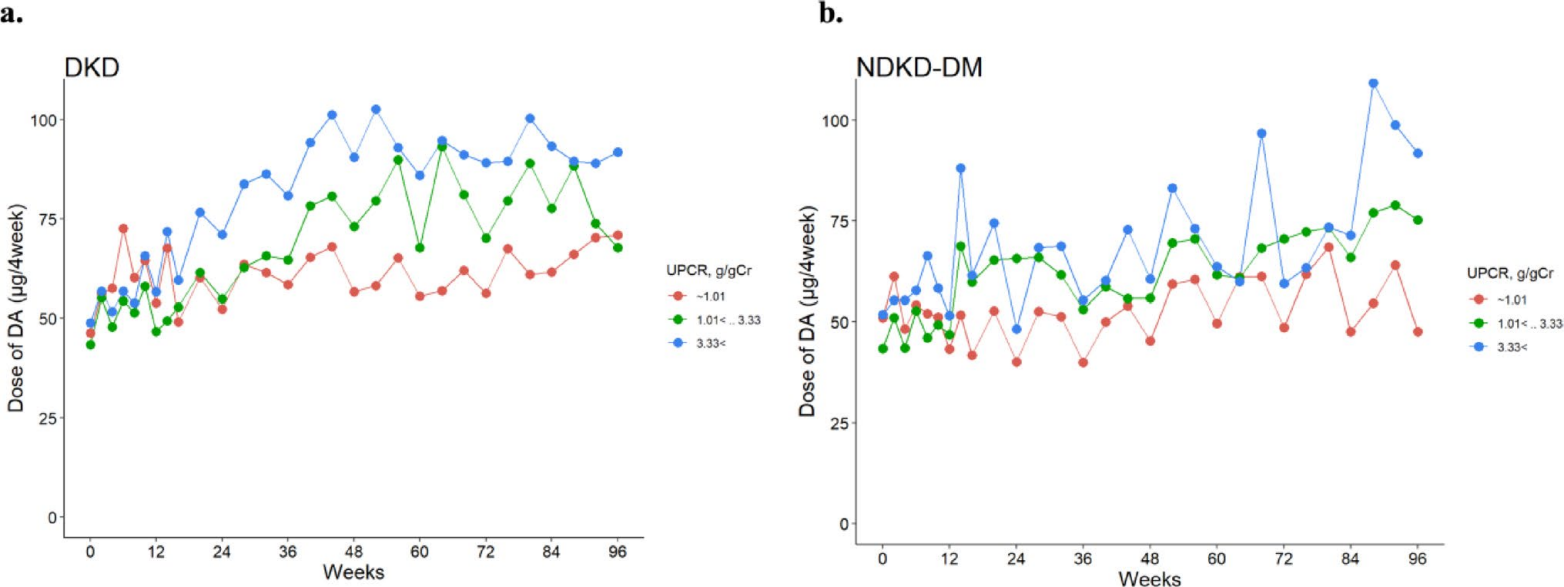
Changes in ERI-1B and DA dosage between the DKD and NDKD-DM groups Change in **a** ERI-1B levels and **b** dosagee of DA administered in the DKD and NDKD-DM groups *DKD* diabetic kidney disease, *NDKD-DM* non-DKD nephropathy with diabetes mellitus, *DA* darbepoetin alfa, *ERI* erythropoietin resistance index, *ESA* Erythropoiesis-stimulating agent

Temporal changes in ERI-1B and DA dosage in the DKD and NDKD-DM groups are shown in Fig. 2. ERI-1B levels increased in the DKD group over time, and the same result was observed with the DA dosage.

### Comparison by CKD stage classification

The clinical characteristics of the DM and non-DM groups according to CKD stage are shown in Table 3, and the clinical characteristics of the DKD and NDKD-DM groups are shown in Table 4. R Regardless of the presence or absence of diabetes, the UPCR increased as the CKD stage progressed. In comparisons of the same CKD stage, no significant difference was observed in Hb or ferritin levels between the DM and non-DM groups or between the DKD and NDKD-DM groups.

**Table 3:** Clinical characteristics and laboratory findings by CKD grade in the DM and non-DM groups

	DM (DKD + NDKD-DM)			non-DM			*P*-value
	G3 (*n* = 90)	G4 (*n* = 295)	G5 (*n* = 223)	G3 (*n* = 108)	G4 (*n* = 378)	G5 (*n* = 309)	
Age, year	70.2 ± 11.4	69.8 ± 11.4	67.5 ± 10.9	73.5 ± 11.4	71.0 ± 12.6	69.2 ± 11.9	NA
Male sex (%)	55.6	66.8	66.4	48.1	53.4	54.7	< 0.001*
BMI, kg/m^2^	23.1 ± 4.0	24.7 ± 4.4	24.9 ± 4.4	21.3 ± 3.0	22.0 ± 3.4	22.8 ± 4.0	NA
BW, kg	57.7 ± 11.5	63.4 ± 12.9	64.5 ± 14.1	52.2 ± 10.2	55.0 ± 10.5	57.9 ± 12.2	NA
Etiology of chronic kidney disease							< 0.001*
Diabetic nephropathy (%)	56.7	63.4	68.6	0.0	0.0	0.0	
Nephrosclerosis (%)	7.8	13.2	9.0	29.6	38.6	33.7	
Chronic glomerulonephritis (%)	15.6	11.5	8.1	27.8	30.2	32.0	
Others (%)	20.0	11.9	14.3	42.6	31.2	34.3	
Current smoker (%)	5.6	16.3	17.0	8.3	4.8	9.7	< 0.001*
Medication							
RAS inhibitors (%)	71.1	72.2	70.0	60.2	64.8	64.7	0.071
Iron supplementation (%)	14.4	15.3	17.0	18.5	15.9	13.6	0.926
Past history							
HT (%)	95.6	98.6	98.2	88.0	94.4	96.8	< 0.001*
DL (%)	60.0	70.2	66.8	46.3	53.2	50.8	< 0.001*
Malignant tumor (%)	18.9	13.2	9.4	13.0	12.4	12.6	0.575
SBP, mmHg	133.1 ± 20.0	136.3 ± 19.6	136.3 ± 19.4	130.5 ± 18.2	131.9 ± 18.8	135.4 ± 17.9	NA
DBP, mmHg	69.0 ± 11.3	69.5 ± 11.6	70.4 ± 12.7	68.9 ± 11.3	71.6 ± 12.9	74.9 ± 12.2	NA
Hb, g/dL	10.0 ± 0.9	9.8 ± 0.8	9.6 ± 0.9	10.0 ± 0.7	9.9 ± 0.9	9.6 ± 0.9	NA
Serum creatinine, mg/dL	1.33 ± 0.27	2.36 ± 0.51	4.23 ± 1.03	1.31 ± 0.26	2.29 ± 0.55	4.19 ± 1.31	NA
eGFR, mL/min/1.73m^2^	38.1 ± 6.4	21.3 ± 4.0	11.4 ± 2.3	37.5 ± 6.5	21.2 ± 4.2	11.3 ± 2.4	NA
Alb, g/dL	3.62 ± 0.52	3.58 ± 0.57	3.63 ± 0.54	3.76 ± 0.46	3.77 ± 0.49	3.87 ± 0.45	NA
HbA1c (NGSP), %	6.67 ± 1.04	6.49 ± 0.98	6.12 ± 0.89	5.66 ± 0.43	5.60 ± 0.40	5.54 ± 0.30	NA
Ferritin, ng/mL	136.4 ± 143.7	149.7 ± 156.6	142.3 ± 124.5	134.2 ± 1689.0	124.6 ± 111.0	126.3 ± 113.8	NA
TSAT, %	23.2 ± 10.5	25.8 ± 10.4	26.6 ± 8.6	26.8 ± 12.5	27.2 ± 9.6	27.7 ± 8.7	NA
NT-proBNP (pg/mL)	31.00 (124.50–638.00)	308.00 (145.50–822.00)	661.00 (342.00–1690.00)	191.00 (106.50–456.00)	281.00 (199.00–1037.50)	500.00 (260.75–1050.00)	NA
UPCR, g/gCr	1.89 ± 3.03	3.06 ± 3.68	4.03 ± 3.63	1.01 ± 2.47	1.43 ± 2.01	2.29 ± 2.86	NA
hsCRP, ng/mL	1400.00 (307.00–2350.00)	1003.00 (218.00–2825.00)	644.00 (260.00–1900.00)	514.00 (226.50–1615.00)	510.00 (177.25–2565.00)	537.50 (198.75–1472.50)	NA

Clinical characteristics and laboratory findings by CKD grade in the DM and non-DM groups. Categorical variables are presented as numbers (percentages). Data are presented as mean ± standard deviation, median (25th and 75th percentile), or percentage

*Alb* serum albumin, *CKD* chronic kidney disease, *DM* Diabetes mellitus, *DKD* diabetic kidney disease, *NDKD-DM* non-DKD nephropathies with Diabetes mellitus, *BMI* body mass index, *BW* body weight, *eGFR* estimated glomerular filtration rate, Hb hemoglobin, HbA1c glycated hemoglobin, *RAS* renin-angiotensin system, *HT* hyper tension, *DL* dyslipidemia, *SBP* systolic blood pressure, *DBP* diastolic blood pressure, *NT-proBNP* N-terminal pro-brain natriuretic peptide, *UPCR* urine total protein to creatinine ratio, *hsCRP* high-sensitivity CRP, *N/A* not available

* P < 0.05

**Table 4:** Clinical characteristics and laboratory findings by CKD grade in DKD and NDKD-DM groups

	DKD			NDKD-DM			*P*-value
	G3 (*n* = 51)	G4 (*n* = 187)	G5 (*n* = 153)	G3 (*n* = 39)	G4 (*n* = 108)	G5 (*n* = 70)	
Age, year	67.1 ± 12.3	68.0 ± 12.2	66.1 ± 10.8	74.3 ± 8.7	72.9 ± 9.2	70.6 ± 10.6	NA
Male sex (%)	58.8	70.6	68.6	51.3	60.2	61.4	NA
BMI, kg/m^2^	23.23 ± 3.64	25.22 ± 4.73	24.76 ± 4.39	22.90 ± 4.33	23.74 ± 3.62	25.33 ± 4.42	NA
BW, kg	58.34 ± 11.32	65.66 ± 13.88	64.83 ± 14.50	56.88 ± 11.89	59.50 ± 9.74	63.73 ± 13.34	NA
Etiology of chronic kidney disease							NA
Diabetic nephropathy (%)	100.0	100.0	100.0	0.0	0.0	0.0	
Nephrosclerosis (%)	0.0	0.0	0.0	17.9	36.1	28.6	
Chronic glomerulonephritis (%)	0.0	0.0	0.0	35.9	31.5	25.7	
Others (%)	0.0	0.0	0.0	46.2	32.4	45.7	
Current smoker (%)	7.8	19.8	19.6	2.6	10.2	11.4	NA
Medication							
RAS inhibitors (%)	68.6	75.4	71.2	74.4	66.7	67.1	NA
Iron supplementation (%)	13.7	17.6	15.7	15.4	11.1	20.0	NA
Past History							
HT (%)	94.1	99.5	97.4	97.4	97.2	100.0	NA
DL (%)	58.8	74.3	68.0	61.5	63.0	64.3	NA
Malignant tumor (%)	13.7	9.1	7.2	25.6	20.4	14.3	NA
SBP, mmHg	133.6 ± 19.4	137.8 ± 19.2	138.0 ± 20.2	132.5 ± 21.1	133.6 ± 20.2	132.6 ± 17.2	NA
DBP, mmHg	70.0 ± 10.9	70.1 ± 11.9	70.7 ± 12.9	67.7 ± 11.8	68.2 ± 10.9	69.7 ± 12.3	NA
Hb, g/dL	9.9 ± 1.0	9.7 ± 0.8	9.6 ± 0.9	10.1 ± 0.9	9.9 ± 0.9	9.6 ± 0.9	NA
Serum creatinine, mg/dL	1.33 ± 0.28	2.41 ± 0.51	4.25 ± 1.02	1.34 ± 0.26	2.27 ± 0.49	4.17 ± 1.05	NA
eGFR, mL/min/1.73m^2^	39.2 ± 7.1	21.2 ± 4.0	11.5 ± 2.3	36.6 ± 4.8	21.4 ± 3.9	11.3 ± 2.2	NA
Alb, g/dL	3.62 ± 0.54	3.51 ± 0.57	3.59 ± 0.55	3.61 ± 0.51	3.71 ± 0.55	3.72 ± 0.50	NA
HbA1c (NGSP), %	6.85 ± 1.17	6.58 ± 1.09	6.17 ± 0.98	6.41 ± 0.74	6.30 ± 0.68	6.00 ± 0.61	NA
Ferritin, ng/mL	144.8 ± 124.0	149.91 ± 156.2	137.5 ± 123.8	126.0 ± 166.2	149.4 ± 158.0	152.7 ± 126.3	NA
TSAT, %	24.0 ± 9.6	25.6 ± 9.9	26.3 ± 8.5	22.1 ± 11.6	26.3 ± 11.2	27.3 ± 8.8	NA
NT-proBNP (pg/mL)	196.50 (83.00–672.25)	251.00 (141.50–404.00)	662.00 (342.50–1695.00)	310.00 (173.00–484.00)	771.00 (316.50–1160.00)	657.00 (334.50–1625.00)	NA
UPCR, g/gCr	2.08 ± 2.59	3.54 ± 3.70	4.50 ± 3.68	1.65 ± 3.55	2.24 ± 3.49	2.99 ± 3.31	NA
hsCRP, ng/mL	1675.00 (347.00–2400.00)	732.00 (311.50–3135.00)	554.00 (223.50–1765.00)	1150.00 (294.00–2320.00)	1710.00 (167.50–2230.00)	900.50 (287.25–2100.00)	NA

Clinical characteristics and laboratory findings by CKD grade in the DKD and NDKD-DM groups. Categorical variables are presented as numbers (percentages). Data are presented as mean ± standard deviation, median (25th and 75th percentile), or percentage

*Alb* serum albumin, *CKD* chronic kidney disease, *DM* Diabetes mellitus, *DKD* diabetic kidney disease, *NDKD-DM* non-DKD nephropathy with diabetes mellitus, *BMI* body mass index, *BW* body weight, *BP* blood pressure, *eGFR* estimated glomerular filtration rate, *Hb* hemoglobin, *HbA1c* glycated hemoglobin, *RAS* renin-angiotensin system, *HT* hyper tension, *DL* dyslipidemia, *SBP* systolic blood pressure, *DBP* diastolic blood pressure, *NT-proBNP* N-terminal pro-brain natriuretic peptide, *UPCR* urine total protein to creatinine ratio, *hsCRP* high-sensitivity CRP, *N/A* not available

**Fig. 3:**
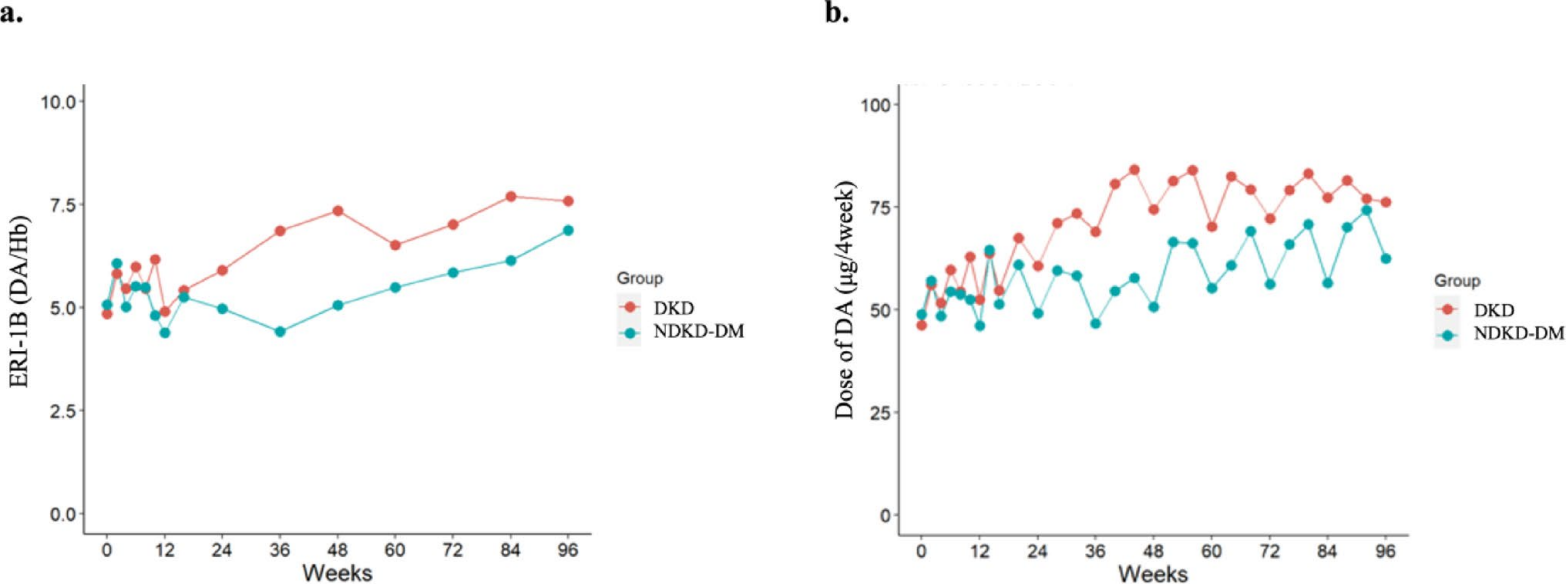
Changes in ERI-1B and DA dosage by CKD grade in the DM and non-DM groups ERI-1B scores for CKD grades 3, 4, and 5 are shown in **a–c**, respectively. Similarly, the DA dosages for CKD grades 3, 4, and 5 are shown in **d–f**, respectively *DM* Diabetes mellitus, *DA* darbepoetin alfa, *ESA* Erythropoiesis-stimulating agent, *CKD* chronic kidney disease

**Fig. 4:**
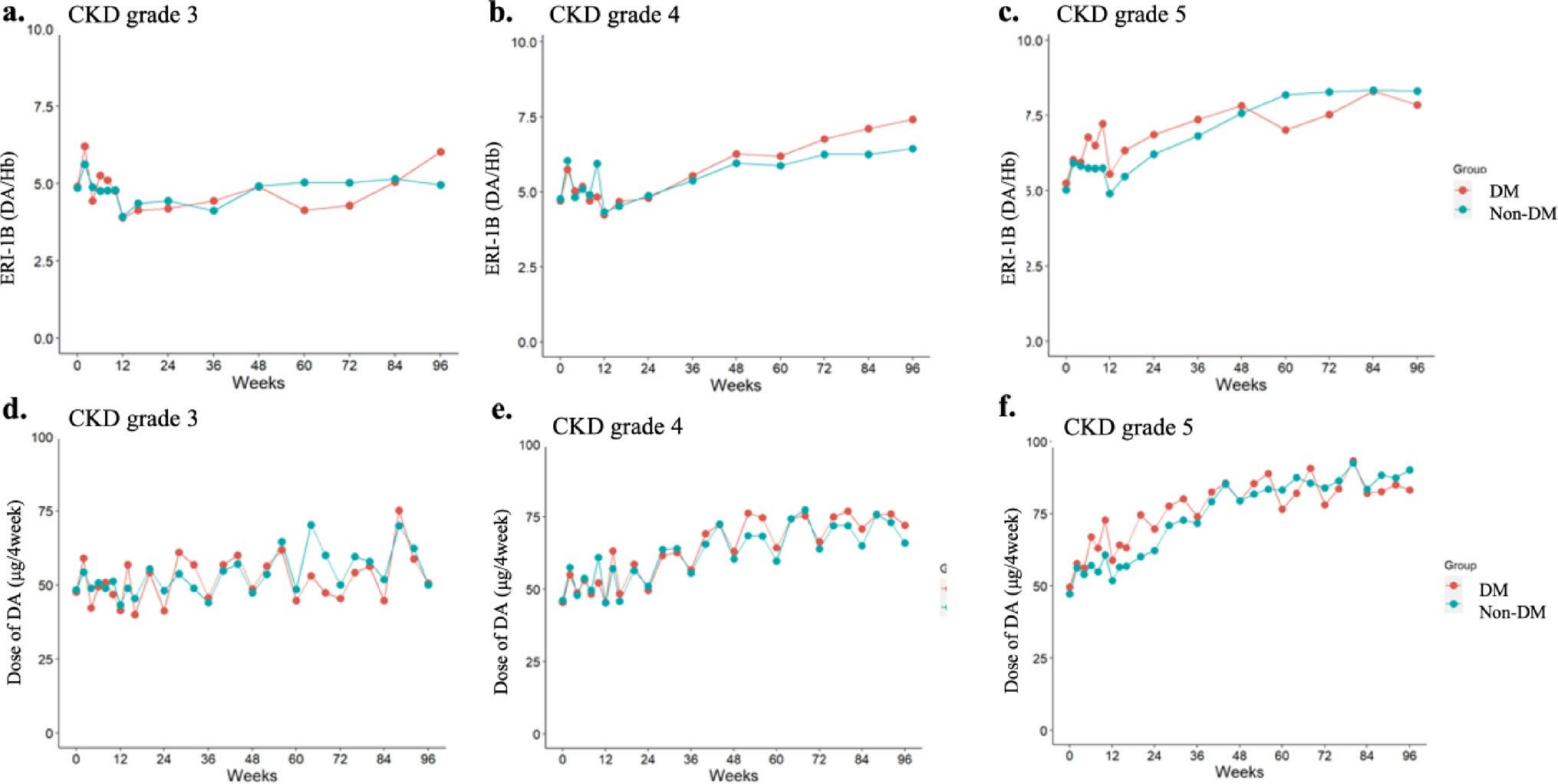
Changes in ERI-1B and DA dosage by CKD grade in the DKD and NDKD-DM groups ERI-1B scores for CKD grades 3, 4, and 5 are shown in **a–c**, respectively. Similarly, the DA dosages for CKD grades 3, 4, and 5 are shown in **d–f**, respectively *DKD* diabetic kidney disease, *NDKD-DM* non-DKD nephropathy with diabetes mellitus, *DA* darbepoetin alfa, *ESA* Erythropoiesis-stimulating agent, *CKD* chronic kidney disease

Overall, the ERI-1B and DA dosage increased with the progression of renal dysfunction; however, no significant difference was observed between patients with and without a history of diabetes (Fig. 3). Compared to the NDKD-DM group, the DKD group required higher ERI-1B and DA dosage at CKD G4 (Fig. 4).

### Stratified analysis by proteinuria level

**Fig. 5:**
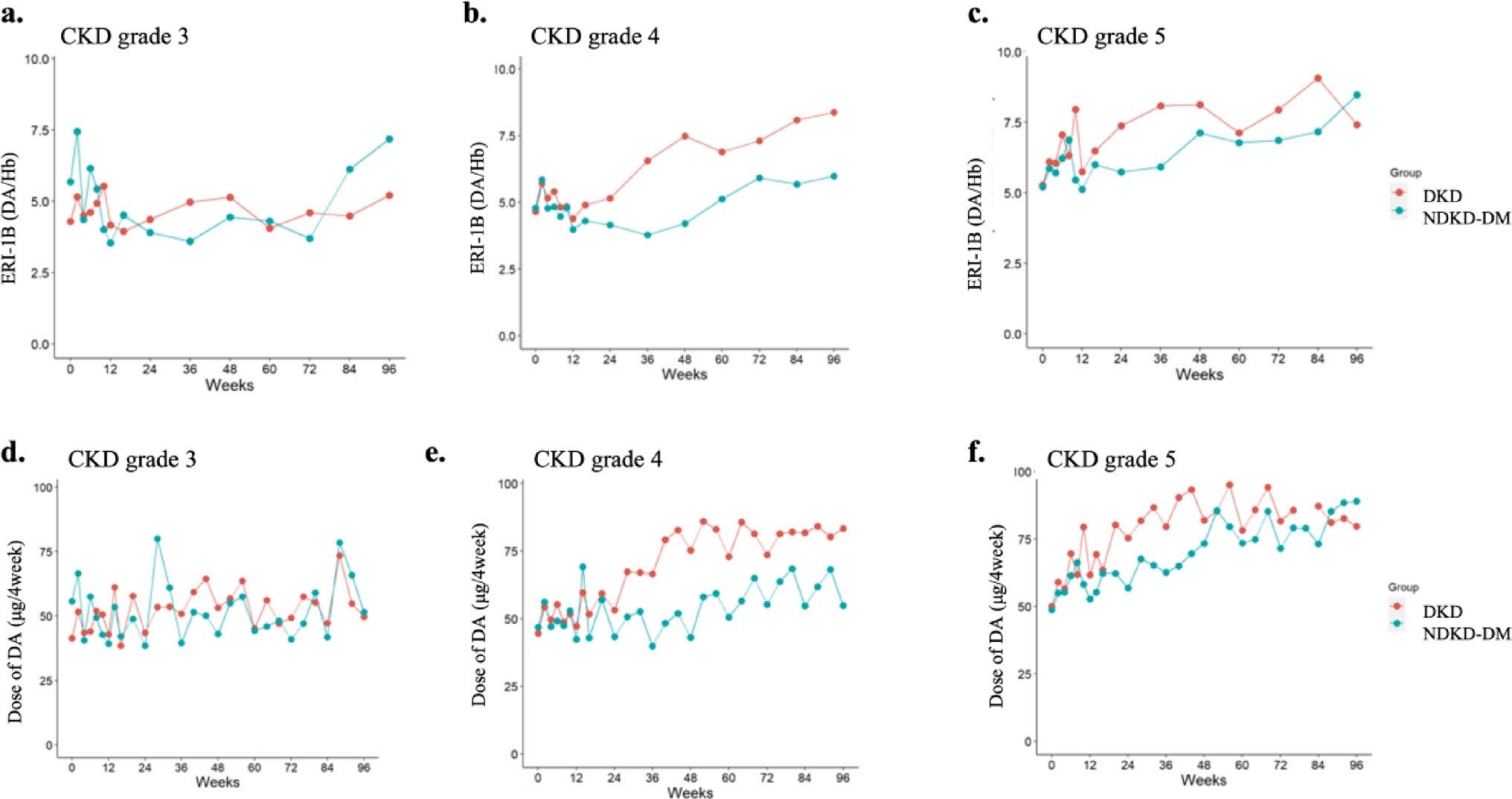
Changes in ERI-1B according to proteinuria levels in the DKD group and NDKD-DM group Change in ERI-1B by proteinuria levels by tertile in the **a** DKD group and **b** NDKD-DM groups *DKD*, diabetic kidney disease, *NDKD-DM* *non-DKD* nephropathy with diabetes mellitus, *ERI* erythropoietin resistance index

**Fig. 6:**
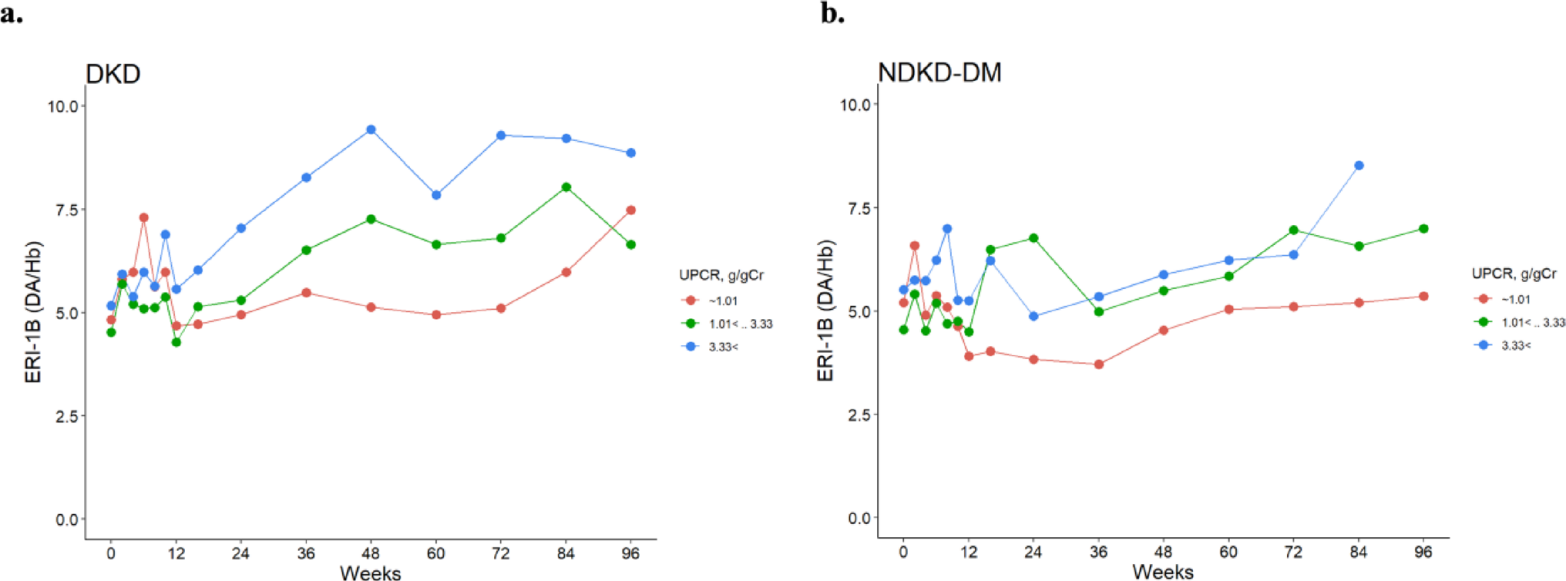
Changes in DA dosage according to proteinuria levels in the DKD group and NDKD-DM group Change in DA dosage by proteinuria levels by tertile in the **a** DKD group and **b** NDKD-DM groups *DKD* diabetic kidney disease, *NDKD-DM* non-DKD nephropathy with diabetes mellitus, *DA* darbepoetin alfa

Next, we compared proteinuria levels by tertile in the DKD group and the NDKD-DM group, respectively. As shown in Fig. 5, ERI-1B tended to increase significantly in the group with higher proteinuria in the DKD group, however, in the NDN-DM group, ERI-1B did not increase in proportion to the increase in proteinuria as seen in the DKD group. Similarly, regarding DA dosage, the DKD group showed a tendency for dosage to increase as urinary protein levels rose, whereas no such result was observed in the NDKD-DM group (Fig. 6).

### Longitudinal changes in ERI-1B and DA dosage

Longitudinal changes in ERI-1B and DA dosage after week 12 were evaluated using a mixed-effects model for repeated measures (MMRM), adjusted for age, sex, body weight, smoking status, history of malignancy, and baseline ERI-1B. Adjusted mean ERI-1B and DA dosage are summarized in Table 5, and the findings were consistent with those of the unadjusted analyses. There were no significant differences in longitudinal changes in ERI-1B or DA dosage between the DM and non-DM groups. However, significant differences were observed between the DKD and NDKD-DM groups.

**Table 5:** The comparison of average ERI-1B and DA dosage after Week 12

Parameter	Comparison group	Analysis model	Group	LSMean	95%Cl			
					Lower	Upper	Difference	*P*-value
ESA-1B (DA dosage/ Hb)	DM group vs non-DM group	Non-Adjusted	DM	6.53	6.10	6.95	0.30	0.288
			non-DM	6.23	5.87	6.59		
		Adjusted^#^	DM	6.47	6.03	6.90	0.21	0.471
			non-DM	6.26	5.89	6.62		
	DKD group vsNDKD-DM group	Non-Adjusted	DKD	6.90	6.40	7.40	1.36	0.002*
			NDKD-DM	5.54	4.86	6.21		
		Adjusted^#^	DKD	6.98	6.46	7.49	1.48	0.001*
			NDKD-DM	5.50	4.81	6.19		
Dosage of DA (mg/4weeks)	DM group vs non-DM group	Non-Adjusted	DM	65.6	62.4	68.8	1.1	0.609
			non-DM	64.5	61.8	67.2		
		Adjusted^#^	DM	64.8	61.6	68.0	0.0	0.993
			non-DM	64.8	62.1	67.5		
	DKD group vsNDKD-DM group	Non-Adjusted	DKD	71.7	67.8	75.5	17.0	p<0.001*
			NDKD-DM	54.6	49.6	59.6		
		Adjusted^#^	DKD	72.3	68.3	76.2	17.8	p<0.001*
			NDKD-DM	54.5	49.3	59.6		

ERI-1B and DA dosage after 12 weeks were compared by using a mixed-effects model with repeated measure with time point, disease group, and interaction with time point and disease group as fixed effects and the baseline value as covariate and patient as the mixed-effect

*DKD* diabetic kidney disease, *NDKD-DM* non-DKD nephropathy with diabetes mellitus, *DA* darbepoetin alfa, *ERI* erythropoietin resistance index, *ESA* Erythropoiesis-stimulating agent

* P < 0.05, ^#^Adjusted were made by adding Age, Sex, Body Weight, Prevalence of smoking and prevalence of malignancy as covariates to the model

## Discussion

In this post hoc analysis of the BRIGHTEN study, when patients with diabetes were divided into DKD and NDKD-DM groups, significant differences in ESA resistance and DA dosage were observed over time. Furthermore, as CKD progressed, ESA resistance increased. In contrast, no significant differences were observed in resistance to ESA or DA dosage between patients with and without diabetes. This study suggests that the development of DKD, rather than the mere presence of diabetes, contributes to the acquisition of ESA resistance.

Diabetes is closely linked to ESA resistance through several mechanisms [4]. One hypothesis is that diabetes induces low-grade chronic inflammation, increasing inflammatory cytokines, such as interleukin-6 and tumor necrosis factor-α, which suppress erythropoiesis. Additionally, inflammation may elevate hepcidin levels, disrupt iron metabolism, and reduce ESA efficacy [13]. Other hypotheses suggest that persistent hyperglycemia increases oxidative stress and shortens red blood cell lifespan and that glucotoxicity affects red blood cell progenitor cells in the bone marrow, directly inhibiting red blood cell production [14]. However, in this study, there was no difference in ESA resistance or DA dosage over the 96-week period, regardless of diabetes status. We also analyzed the data by stratifying patients according to CKD stage, where urinary protein levels increased. However, ESA resistance did not differ between patients with and without diabetes (Fig. 3). Therefore, other factors beyond diabetes may contribute to ESA resistance.

The Kidney Disease Outcomes Quality Initiative of the US Renal Foundation defines DKD as CKD in which diabetes is involved in the onset and progression of the disease, without requiring a pathological diagnosis [15]. If no clear evidence suggests kidney disease due to a cause other than diabetes and CKD is present, the condition is clinically diagnosed as DKD. Although the concept of NDKD-DM partially overlaps with that of DKD, it is assumed that the effects of diabetic kidney damage are only partially involved compared to DKD. In the NDKD-DM group, chronic glomerulonephritis and nephrosclerosis were observed in 35.6% and 30.5% of patients, respectively. Although diabetes was present as a comorbidity, the primary cause of renal dysfunction was assumed to be unrelated to diabetes. A difference in ESA resistance was observed between the DKD and NDKD-DM groups **(**Fig. 4; Table 5**)**. The presence of DKD, rather than diabetes itself, has been suggested to be associated with ESA resistance.

Notably, HbA1c levels were higher in the DKD group than in the NDKD-DM group. However, since both groups had diabetes, the effect of hyperglycemia on ESA resistance was assumed to be minimal. Chronic inflammation contributes to ESA resistance by increasing inflammatory cytokines, and it also causes iron utilization disorders [4, 13]. However, the high-sensitivity CRP levels, which suggest inflammation, and ferritin level and TSAT, which suggest iron status, were not higher in the DKD group than in the NDKD-DM group. Thus, variations in ESA resistance may be partly explained by differences in the qualitative characteristics of proteinuria between DKD and NDKD-DM [16, 17]. Furthermore, differences in ESA resistance may result from differences in the underlying diseases that contribute to renal damage in patients with DKD and NDKD-DM. Proteinuria serves as both a marker and driver of chronic inflammation, and its presence is strongly associated with ESA resistance. There was a difference in the amount of urinary protein between the DKD and NDKD-DM groups, which was thought to be due to differences in the diseases affecting renal damage. Furthermore, stratified analysis by proteinuria level showed a tendency for ESA resistance to increase with rising proteinuria in the DKD group, whereas no such trend was observed in the NDKD-DM group (Fig. 5). These findings indicate that the quality of proteinuria differs between DKD and NDKD-DM groups, suggesting that proteinuria in the DKD group may be associated with ESA resistance.

Several interrelated pathophysiological processes contribute to the development of proteinuria in DKD. The mechanisms are thought to be as follows [18]: (1) advanced glycation end products accumulate, thickening and stiffening the glomerular basement membrane (GBM), reducing its selective barrier function; (2) high blood glucose levels damage podocytes, causing gaps to appear in the filtration barrier; (3) hyperfiltration due to altered hemodynamics causes albumin leakage; and (4) excessive protein filtration leads to tubular inflammation and injury [19]. Meanwhile, the mechanisms underlying the development of proteinuria in chronic glomerulonephritis and nephrosclerosis are thought to be as follows [20–22]: (1) immune complexes in the GBM or mesangium trigger complement activation and inflammation, increasing the permeability of capillaries; (2) hypertension causes thickening and narrowing of afferent arterioles, reducing blood flow to the glomerulus; (3) long-term ischemia causes scarring of the glomerulus, reducing its filtration capacity and changing its barrier function. Unfortunately, the BRIGHTEN study did not measure albuminuria, making it impossible to accurately assess differences in proteinuria quality. However, in DKD, the effects of hyperglycemia and hemodynamic changes are thought to be more pronounced than those in chronic glomerulonephritis and nephrosclerosis, potentially leading to the production of distinct types of urinary proteins. If kidney dysfunction progresses to a high degree, structural destruction of the glomeruli also progresses in DKD, and proteinuria appears in the same manner as in nephrosclerosis. Therefore, the differences in ESA resistance and DA dosage between DKD and NDKD-DM were thought to be smaller in CKD5 than in CKD4 (Fig. 4**)**. However, this hypothesis warrants further investigation.

The progression of renal impairment was more rapid in the NDKD-DM group than in the DKD group. Therefore, classifying these two diseases is clinically important, even when a kidney biopsy is unavailable. In particular, the treatment strategies for controlling proteinuria differ. To control proteinuria, it is important to control hyperglycemia, oxidative stress, and RAAS activation in DKD, whereas in NDKD-DM, treatment according to the underlying disease is important. Although it was not possible to analyze the treatment content in this study, it is suggested that more aggressive treatments for diabetes, such as the use of SGLT-2 inhibitors, should be considered for controlling ESA resistance in DKD than in NDKD-DM [23–25].

The present study has certain limitations. We collected data from a large nationwide cohort of patients with CKD who required ESA; however, many were clinically rather than pathologically diagnosed. Glomerulonephritis subtypes and diabetes duration were largely unknown, potentially leading to misclassification, despite all physicians being nephrologists. Furthermore, clinically, many cases cannot be completely distinguished between DKD and NDKD-DM, and it is possible that some cases may have been assigned incorrectly. In addition, there were only a limited number of cases with no evidence of kidney dysfunction (CKD stage G1 or 2). Consequently, comparisons with cases exhibiting proteinuria but preserved kidney function were not feasible. Furthermore, this analysis did not consider the progression of kidney dysfunction over time. It is possible that the DKD group showed more rapid kidney dysfunction progression and increased ESA resistance [17]. Further studies that consider kidney dysfunction are required.

In conclusion, although diabetes has generally been considered related to ESA resistance, this study suggests that the development of DKD, rather than the mere presence of diabetes, may contribute to the acquisition of ESA resistance. Although the precise mechanism underlying ESA resistance remains unclear, our findings suggest that it is closely linked to the proteinuria characteristics of DKD. These findings highlight the role of proteinuria and inflammation in the ESA response, emphasizing the need for individualized anemia management strategies based on nephropathy type. Further research is required to elucidate this relationship and develop targeted strategies for managing ESA resistance in patients with DKD.

## Supplementary Information

Below is the link to the electronic supplementary material.

Supplementary Material 1
