# Supplementary material for: Erythropoiesis-stimulating agent resistance is associated with diabetic kidney disease but not with diabetes: a post hoc analysis of the BRIGHTEN study
Source: Clin Exp Nephrol. 2026 May 18;30(8):1196–207. doi: 10.1007/s10157-026-02881-2 (PMC13379487; doi:10.1007/s10157-026-02881-2)
Supplement: Supplementary file 1 — Supplementary Material 1 [file 10157_2026_2881_MOESM1_ESM.docx]

**Supplementary Table 1. Clinical diagnostic methods for nephrosclerosis, diabetic kidney disease, and non diabetic kidney disease with diabetes**

| **1. Diabetic kidney disease (DKD)** |
| --- |
| - CKD patients with possible clinical situation and/or sign (long-standing diabetes [>10 years], diabetic retinopathy, and no or mild kidney atrophy, and proteinuria. - Absence of signs or symptoms of other primary causes of kidney damage. - Absence of glomerular hematuria. |
| **2. Non diabetic kidney disease with diabetes (NDKD+DM)** |
| - CKD patients with presence of glomerular hematuria and/or proteinuria, or biopsy-proven kidney disease other than diabetic kidney disease prior to the onset of diabetes. |

CKD, chronic kidney disease.
